# Encapsulation of Natural Polyphenolic Compounds; a Review

**DOI:** 10.3390/pharmaceutics3040793

**Published:** 2011-11-04

**Authors:** Aude Munin, Florence Edwards-Lévy

**Affiliations:** Institute of Molecular Chemistry of Reims, Faculty of Pharmacy of Reims, University of Reims Champagne-Ardenne, 51 rue Cognacq-Jay, 51100 Reims, France

**Keywords:** polyphenol, antioxidant, free radical scavenger, encapsulation

## Abstract

Natural polyphenols are valuable compounds possessing scavenging properties towards radical oxygen species, and complexing properties towards proteins. These abilities make polyphenols interesting for the treatment of various diseases like inflammation or cancer, but also for anti-ageing purposes in cosmetic formulations, or for nutraceutical applications. Unfortunately, these properties are also responsible for a lack in long-term stability, making these natural compounds very sensitive to light and heat. Moreover, polyphenols often present a poor biodisponibility mainly due to low water solubility. Lastly, many of these molecules possess a very astringent and bitter taste, which limits their use in food or in oral medications. To circumvent these drawbacks, delivery systems have been developed, and among them, encapsulation would appear to be a promising approach. Many encapsulation methods are described in the literature, among which some have been successfully applied to plant polyphenols. In this review, after a general presentation of the large chemical family of plant polyphenols and of their main chemical and biological properties, encapsulation processes applied to polyphenols are classified into physical, physico-chemical, chemical methods, and other connected stabilization methods. After a brief description of each encapsulation process, their applications to polyphenol encapsulation for pharmaceutical, food or cosmetological purposes are presented.

## 1. Introduction

Polyphenols are secondary metabolites present in all vascular plants, and constitute a large family of ubiquitous and varied substances, from simple molecules to complex structures. These natural substances all have in common the presence of one or several benzenic cycles bearing one or several hydroxy functions and deriving from the metabolism of shikimic acid and/or polyacetate [1–3].

To date, several thousands of polyphenolic compounds have been characterized in plants, and grouped together in various classes. Inside each of these classes, the variations around the basic chemical skeleton essentially concern the degrees of oxidation, hydroxylation, methylation, glycosylation and the possible connections to other molecules (primary metabolites such as carbohydrates, lipids, proteins, or phenolic secondary metabolites for example).

During evolution, adaptation of living species to oxygen was achieved by the appearance of enzymes facilitating not only its consumption, but also the detoxification of its highly reactive metabolites, the Reactive Oxygen Species (ROS). In 1956, Harman proposed the free radical theory of ageing according to which a dysfunction of the oxygen regulation system would induce oxidative damage to biomolecules which, on accumulation, would be responsible for the ageing process [4,5]. When the capacity of the body to detoxify ROS is exceeded, oxidative stress occurs as the result of a pronounced imbalance between pro-oxidant and antioxidant effects [6]. Nowadays, the implication of this phenomenon of cell aggression in numerous pathologies is widely demonstrated.

Free radical damage appears to be partially limited by the action of natural antioxidant compounds present in daily food, namely polyphenols. Besides the specific properties of some classes, they all share two fundamental properties which participate in their antioxidant capacities: interaction with proteins or with ions, and radical scavenging activity. Polyphenols can act using different modes of action: by molecular complexation with pro-oxidant proteins, by chelation of potentially pro-oxidant metal ions (Fe^3+^, Al^3+^, Cu^2+^) or by direct trapping of ROS [7].

Among their properties, the strong antioxidant power of polyphenols is probably the most documented [7–17]. Numerous *in vitro* studies have demonstrated that polyphenolic compounds can directly scavenge molecular species of active oxygen such as superoxide radical (O_2_^•^), hydrogen peroxide (H_2_O_2_), hydroxyl radical (HO^•^), singlet oxygen (^1^O_2_) or peroxyl radicals (RO_2_^•^). Indeed, polyphenols possess ideal structural features for their antioxidant action, mainly due to their ability to donate hydrogen atoms (1) or electrons (2) [7–9,12,13].

(1) $$\begin{array}{cc} \text{H-atom}\;\text{transfert}\;\left(\text{HAT}\right) & {\text{X}}^{•}\;+\;\text{ArOH}\;\to \text{XH}\;{+\;\text{ArO}}^{•} \end{array}$$

(2) $$\begin{array}{cc} \text{Single-electron}\;\text{transfert}\;\left(\text{SET}\right) & {\text{X}}^{•}\;+\;\text{ArOH}\to {\text{X}}^{-}\;+\;{\text{ArOH}}^{•+} \end{array}$$

In the Hydrogen-Atom Transfert (HAT) mechanism, the phenolic antioxidant (ArOH) reacts with the free radical (X^•^) and becomes a free radical (ArO^•^) by transferring a hydrogen atom through homolytic rupture of the O-H bond. The ease of formation and stability of ArO^•^ is strongly dependent upon the structural features of the ArOH compound. The most important determining factors are the presence, number, and relative positions of additional phenolic hydroxy groups, their involvement in the formation of intramolecular hydrogen bonds, and the conformationally dependent possibility of allowing electronic delocalization throughout the largest part of the molecule. All of these factors affect the dissociation energy (BDE) of the phenolic O-H bond: the weaker the O-H bond, the easier the H-atom transfer will be.

The second mechanism is the Single-Electron Transfer (SET) from ArOH to a free radical X^•^ with formation of a stable radical cation ArOH^•+^. The ionization potential (IP) of ArOH is thus another important physicochemical parameter for assessing the antioxidant efficacy of plant polyphenols: the lower the IP, the easier the one-electron transfer is.

The BDE and the IP of the polyphenol are the two basic physicochemical parameters that can be used to determine the potential efficacy of each mechanism, respectively.

The stabilization of the resulting phenoxy radicals, ArOH^•+^ and ArO^•^, is a result of the delocalization of their unpaired electron over the aromatic ring by resonance or by hyperconjugation effects.

Furthermore, the high tendency of polyphenols to chelate metal ions may contribute to their antioxidant activity by preventing redox-active transition metals from catalyzing free radical formation [7]. Indeed, polyphenols may inactivate iron ions by chelation and consequently suppress the superoxide-driven Fenton reaction, with is believed to be the most important source of harmful ROS. Flavonoids have been widely reported to chelate metals, and potential metal-binding sites have been identified. Indeed, polyphenolic compounds possess hydroxyl and carboxyl groups able to bind metal ions bearing strong positive charges such as iron (III) and copper (II). For chelation, bidentate ligands are much more powerful scavengers towards metal cations than monodentate ligands. The protonated phenolic group is not a good ligand for metal cations, but once deprotonated, an oxygen center is generated that possesses a high charge density. Furthermore, the metal-chelating ability of polyphenols could also be related to the high nucleophilic character of the aromatic rings rather than to specific chelating groups within the molecule.

However, the plethora of health benefits reported in the scientific literature also results from the capacity of polyphenols to interact with proteins (enzymes, membrane receptors, tissue proteins) in a specific way, thus allowing them to protect or modulate their activity [18–37]. Polyphenols act as potent inhibitors of ROS-generating enzymes such as xanthine oxydase [31], cyclooxygenase and lipoxygenase [32], by complexing the protein. The process of polyphenol complexation is directly influenced by the protein characteristics (solubility, molecular mass, hydrodynamic volume, isoelectric point and amino-acid composition) [18,22,25,28] and the polyphenol characteristics (molecular mass, structure, conformational flexibility, water solubility) [23,25,28,34,37]. The physicochemical conditions (pH, nature of the solvent, temperature, ionic strength, presence of other organic molecules such as polysaccharides) must also be taken into account [19–22,25,28–30]. The main types of interactions involved in the complexation mechanism are non-covalent bond formation and hydrophobic interactions [34].

The literature (Table 1) shows that, *in vitro* and/or *in vivo*, polyphenols are able to:

- reduce the inflammation by inhibition of the edema,
- stop the development of tumors,
- present proapoptotic and anti-angiogenic actions,
- modulate the immune system,
- prevent the osseous disturbances incriminated in the osteoporosis,
- increase the capillary resistance by acting on the constituents of blood vessels,
- protect the cardiovascular system,
- protect the retina,
- limit weight gain…

The economic implications of polyphenolic compounds are thus substantial. They are used in numerous sectors of the food-processing industry as natural additives (natural coloring agents, conservative agents, natural antioxidants, nutritional additives). However, it is probably in the field of human health that the economic implication of polyphenols is the most important. Actually, many plant extracts rich in phenolic molecules of interest are used as food complements or can be integrated into cosmetic or pharmaceutical formulations.

Unfortunately, these valuable natural compounds's uses are substantially limited [38]. It is reported that the polyphenol concentrations needed to obtain *in vitro* efficiency are generally superior to *in vivo* moderate levels. According to the route of administration, the efficiency of these compounds depends on their bioavailability and integrity. Indeed, a small proportion of molecules administered orally are absorbed, because of insufficient gastric residence time, low permeability and/or low solubility. Their instability during food processing, distribution or storage, or in the gastrointestinal tract (pH, enzymes, presence of other nutrients), limits the activity and the potential health benefits of polyphenols. The topical use of natural polyphenols is also delicate because of their important sensitivity to environmental factors, including physical, chemical, and biological conditions. Unfortunately, they oxidize very quickly, leading to the progressive appearance of a brown color and/or unwanted odors with a considerable loss in activity.

Other problems related to polyphenol use in human health have to be solved. A large number of polyphenolic compounds from natural sources are interesting for their properties, however in their freeform, they can show limited water solubility. Furthermore, many polyphenols have an unpleasant taste which must be masked before their incorporation in foodstuffs or oral medicines.

Therefore, the administration of phenolic compounds requires the formulation of a finished protecting product able to maintain the structural integrity of the polyphenol until the consumption or the administration, mask its taste, increase its water solubility and bioavailability, and convey it precisely towards a physiological target.

Among the existing stabilization methods, encapsulation is an interesting means. The use of encapsulated polyphenols instead of free compounds is the source of numerous works.

Nowadays, various microencapsulation techniques are available [41–44], and the microencapsulated products are widely used in the food, pharmaceutical and cosmetic industries, but also in various other domains like personal care, agricultural products, veterinary medicine, industrial chemicals, biotechnology, biomedical and sensor industries. The particles obtained are called microcapsules or microspheres according to the internal structure, core-shell-like or matrix, respectively. Microparticles may contain a solid, liquid or gaseous active substance, with a size range between about 1 micron and 1 millimeter. Particles with a smaller size, from 1 nanometer to 1 micrometer, are called nanoparticles, and nanocapsules and nanospheres can also be distinguished according to their internal structure.

The coating materials include polymers of natural or synthetic origin, or lipids.

According to the needs related to a specific field of application, the particles are elaborated to perform the following functions:

- protect a fragile or unstable compound from its surrounding environment,
- protect the user from the side-effects of the encapsulated compound,
- trap a compound (aromas, organic solvents, pesticides, essential oils …),
- modify the density of a liquid,
- change a liquid into a solid,
- isolate two incompatible compounds that must coexist in the same medium,
- control the release of the encapsulated compound…

There are a very large number of encapsulation methods that can be classified as follows:

- Physical methods: spray-drying, fluid bed coating, extrusion-spheronization, centrifugal extrusion, processes using the supercritical fluids;
- Physicochemical methods: spray-cooling, hot melt coating, ionic gelation, solvent evaporation-extraction, simple or complex coacervation;
- Chemical methods: interfacial polycondensation, *in situ* polymerization, interfacial polymerization, interfacial cross-linking …

This review focuses on the most commonly used encapsulation methods applied to polyphenols, and discusses their effectiveness. Although some remarkable nanoencapsulation results will be presented, encapsulation of natural polyphenols on the micro scale will be the main topic of this article.

## 2. Physical Methods

### 2.1. Spray-Drying

**Figure 1.**
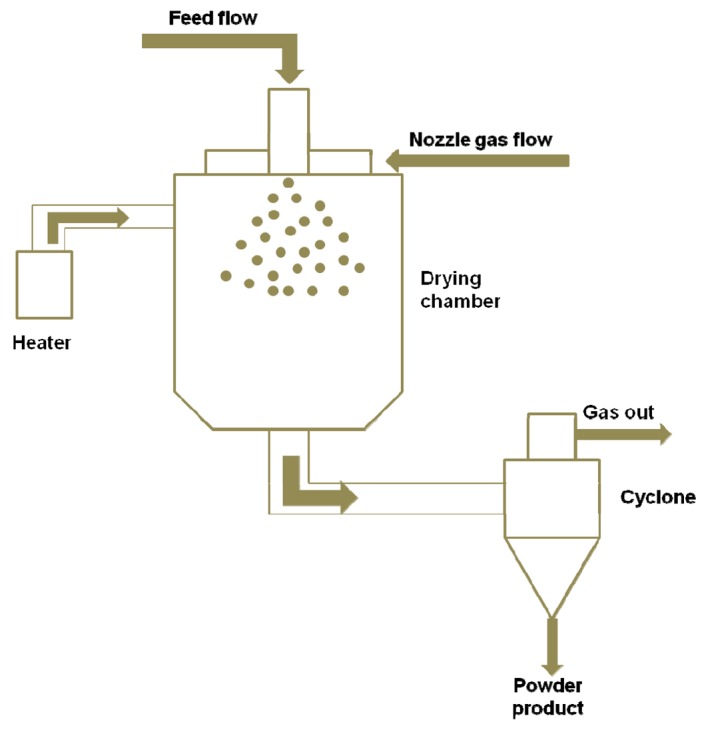
Schematic illustration of a spray-drying apparatus.

The spray drying technique involves a specific apparatus (Figure 1), allowing the formation of particles from a dispersion of active compound in a solution of coating agent [45–48]. First, a liquid formulation containing a coating agent and the active ingredient in a solvent is atomized into droplets via either a nozzle using compressed gas to atomize the liquid feed, or a rotary atomizer using a wheel rotating at high speed. Then, a heated process gas (air or nitrogen) is brought into contact with the atomized feed using a gas disperser, leading to evaporation of the solvent. As the liquid rapidly evaporates from the droplet, a particle forms and falls to the bottom of the chamber. The powder is recovered from the exhaust gases using a cyclone or a bag filter.

Spray drying is a very rapid drying method due to the very large surface area created by the atomization of the liquid feed. It is a single-stage method and the process can be conducted continuously.

This process is widely used in the industry for the production of microspheres or microcapsules, according to the initial nature of the sprayed liquid: solution, suspension or emulsion. The size of the particles obtained is generally around 10 micrometers, with a large size distribution due to variety of droplet sizes in the spray. The most influential parameters are the geometry of the nozzle and the initial solution viscosity. This technique is relatively low cost, flexible, and leads to the production of high quality and stable particles, making this technique the most used in the food industry.

The liquid solution containing the coating agent and the phenolic active substances is transformed according to this process into dry microparticle powders. The most common wall materials are gum arabic, maltodextrin, and modified starch. The resulting particles are more or less spherical, with a size distribution between 10 and 100 micrometers.

A study of the influence of the wall component nature by the technique of spray-drying on the rate of encapsulation was realized during the microencapsulation of an extra virgin olive oil. In optimized conditions, proteins (sodium caseinate and gelatin), hydrocolloids (gum arabic) and hydrolyzed starch (starch, lactose and maltodextrin), were tested as wall materials. A high encapsulation efficiency of some oils (53%) was obtained when the coating agent was gelatin, gum arabic, maltodextrin or sodium caseinate-maltodextrin conjugates [49].

Maltodextrins turned out to be the best thermal defenders, essential to preserve the integrity of the anthocyanins during their encapsulation [50,51].

Nowadays, maltodextrin is commonly mixed with gum arabic. A mixture of maltodextrin (60%) and gum arabic (40%) has been used for encapsulation of procyanidins from grape seeds [52]. No change of procyanidins was observed during the critical drying stage, the rate of encapsulation was around 85 %, and their stability was improved. Epigallocatechin gallate (EGCG) was encapsulated within the same carbohydrate matrix, with the same encapsulation efficiency of 85%. These particles were able to inhibit steps of the tumorigenesis process [53].

**Figure 2.**
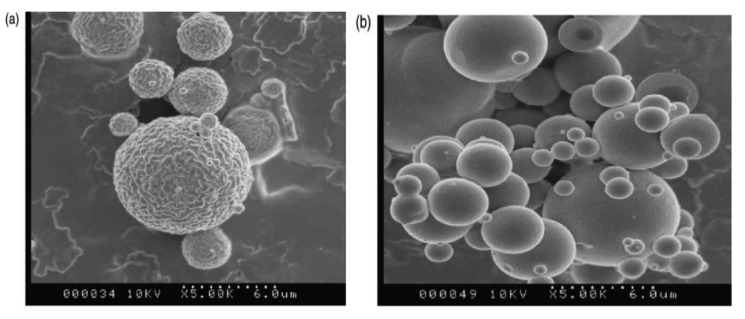
Scanning electron micrographs of (**a**) blank microspheres and (**b**) microspheres loaded with olive tree leaves extract (OLE). Reprinted with permission from Elsevier [54].

Chitosan can be used as a coating material for the encapsulation of olive tree leaves extract (OLE) [54]. Microspheres loaded with OLE (27%) into chitosan, revealed a perfectly smooth surface with regard to the blank microspheres (Figure 2), indicating the influence of structural interactions between polyphenols present in this extract and the matrix polymers.

More recently, a soybean extract rich in polyphenols was immobilized within a matrix composed of maltodextrin, starch or a silica (Tixosil® 333) [55]. The results show that the Tixosil 333 reduced the degradation of the encapsulated polyphenol and protected its antioxidant activity. The addition of this excipient during the drying step guarantees the stability and the efficacy of the finished product. Carrageenan showed to be an interesting material as a means of conservation of the antioxidant activity for the encapsulation of diverse natural polyphenol-rich extracts [56,57].

Another type of material, *i.e.*, protein-lipid systems, showed an important encapsulation efficiency of polyphenolic compounds. Grape seed extract, apple extract and olive tree leaf extract, rich in oleuropein, were immobilized within a sodium caseinate—soy lecithin matrix [58]. Microscopic observations and granulometric analysis revealed the presence of spherical particles, presenting a homogeneous size (80% of particles were 6–60 μm). The preservation of the antioxidant activity, according to the polyphenol concentration after encapsulation by the method of spray-drying was demonstrated. These results demonstrate the retention of the entrapped polyphenols and can be used for nutraceutical application.

The spray-drying technique turned out to be a good method to prepare spheres containing fresh artichoke (*Cynara scolymus*) extract. The studies show the importance of the choice of the excipient (lactose and/or hypromellose) on the morphology of the prepared microspheres and on the *in vitro* release kinetics of the loaded extract. The authors note that this release formulation could be proposed in a nutraceutical controlled release oral dosage form [59].

The encapsulation of an extract of oak (*Quercus resinosa*), very rich in polyphenols, was recently realized by means of a high-pressure homogenization [60]. This extract presents instability, bad taste and strong astringency which require its encapsulation before its incorporation in foodstuffs. Within a matrix consisting of sodium caseinate and lactose, a high antioxidant activity was measured even at very low phenolic concentrations.

The aim of another work was to improve the solubility profile of naringenin in spray-dried particles prepared with alpha-glucosyl hesperidin (Hsp-G). The results suggest the formation of a micelle-like structure in which naringenin was incorporated with Hsp-G molecules by specific molecular interaction resulting in the anomalous enhancement in the solubility of this model hydrophobic polyphenol [61].

Spray-drying is also a stabilizing dehydrating method, which can be used without wall material. A recent work on the thermal stability and photostability of a yerba mate (*Ilex paraguariensis)* spray-dried powder (SDP) showed that SDP was stabilized against ultraviolet C radiation for 48h and for 4 months at 40 °C under an atmosphere of high relative humidity [62].

### 2.2. Encapsulation Processes Using Supercritical Fluids

The available classical encapsulation techniques present several disadvantages. Indeed, they often require a large amount of organic solvents, surfactants and other additives which can lead to emission of volatile organic compounds, raise waste elimination problems, and leave potentially toxic residues contained in finished products. Some techniques result in the preparation of particles having a low loading rate and for which post-treatments, often essential, lengthen the process. Moreover, pH and temperature conditions required for some processes are critical factors limiting their application.

Encapsulation processes using supercritical fluid technology have been developed during these last years. The properties of supercritical fluids are often described as intermediate between those of a liquid and a gas. These properties can be easily changed with variations in pressure and temperature. Carbon dioxide is the most widely used supercritical fluid because of its relatively low critical temperature (Tc = 304.2 K) and pressure (Pc = 7.38 MPa). In particular, its low critical temperature makes it highly suitable for processing heat-sensitive materials. In addition, supercritical CO_2_ (scCO_2_) is non-toxic, non-flammable, inexpensive, and has GRAS status [63].

The processes are generally classified in three families, depending on the way the supercritical fluid is used:

- As a solvent: Rapid Expansion of Supercritical Solutions (RESS) and derived processes;
- As an anti-solvent: Supercritical Anti Solvent (SAS) and derived processes;
- As a solute: Particles from Gas Saturated Solutions (PGSS) and derived processes.

Two of these processes applied to polyphenol encapsulation will be approached in more detail below.

#### 2.2.1. Supercritical Antisolvent Processing

**Figure 3.**
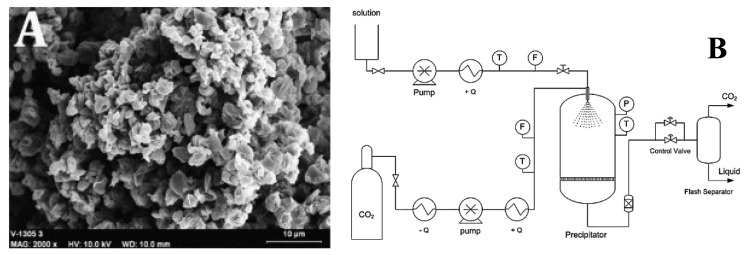
(**A**) SEM micrographs of the green tea extract co-precipitated with polycaprolactone (PCL) (MW: 25,000) by Supercritical Antisolvent Process and (**B**) schematic diagram of the SAS pilot plant. Reprinted with permission from Elsevier [64].

When the solute is very weakly soluble in the supercritical fluid, the latter can be used as antisolvent. The supercritical fluid or supercritical antisolvent is injected into a pressurized container containing the solution (organic solvent + solute to micronize) (Figure 3B). The precipitation cell is partially filled with the solution, whereas the supercritical fluid is brought to a chosen pressure, then introduced into the reactor. In contact with the solution, the supercritical antisolvent dissolves in the phase, decreasing its density and the solvation power of the organic solvent. At the same time, the solvent evaporates in the supercritical phase, leading to the oversaturation of the solution, then to precipitation of the solute. Once these particles have formed, the excess of solvent is eliminated under a continuous flow of pure supercritical fluid via a purge gate. The particle size distribution according to this process is relatively heterogeneous.

By means of a semi-continuous near supercritical antisolvent process, green tea polyphenols were co-precipitated with a biodegradable polymer (*polylactide*-*polycaprolactone copolymer* or PLC) [64]. In this study, the effect of different operating parameters (operating pressure and temperature, concentration ratio polymer/tea extract and antisolvent to solution flow ratio) on the precipitation yield, polyphenol content and particle morphology and size, of the co-precipitated product obtained were studied. The total polyphenol content represented from 60 to 100% of the maximum theoretical composition and the microparticles (3–5 μm) had a homogeneous size distribution and were much agglomerated (Figure 3A). The results of drug release showed sequential kinetics, about 30% of the drug being released during the first 4 h, the remaining 70% being then progressively released in 90 h. The authors suggest that in polycaprolactone (PCL), the fast initial release would be due to diffusion of the drug through the matrix. The remainder of the drug would be released only when the matrix would start degrading.

#### 2.2.2. Gas Saturated Solutions Process

**Figure 4.**
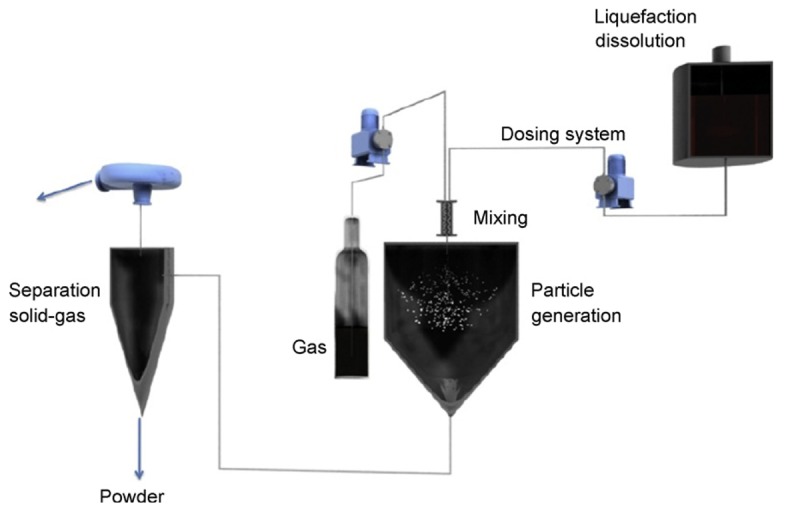
Schematic flowsheet of the particles from gas saturated solutions (PGSS) process. Reprinted with permission from Elsevier [63].

In this process, also known as supercritical-assisted atomization (SAA) process, the supercritical fluid plays the role of a solute because under pressure gases can be dissolved in liquids (Figure 4). The process consists of the solubilization of a large quantity of supercritical fluid in a melted substance, dissolved or dispersed in a solvent. Under these conditions, the gas dissolution into the liquid phase causes the formation of a gas-saturated solution. This last one is then expanded through a nozzle into the atomization chamber, to form solid particles or liquid droplets. With the combined action of the cooling mixture and the volume expansion of gas, the precipitation of the substance begins. The particles are collected in the reactor after depressurization.

**Figure 5.**
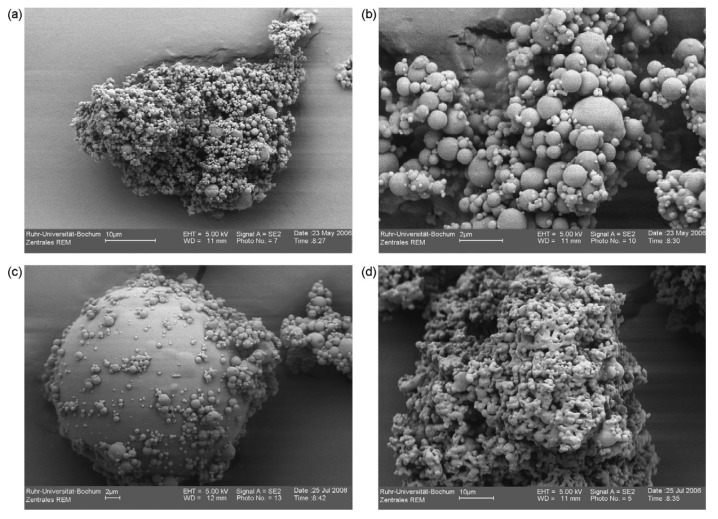
Scanning electron micrographs of the green tea samples produced by PGSS drying process. Reprinted with permission from Elsevier [65].

This new high-pressure process was used for the gentle drying of natural extracts [65]. After optimization of the drying process, initial investigations with this technique led to successful encapsulation of green tea extracts without degradation of the active ingredients. The SEM pictures showed spherical particles with an approximate size of around 10 μm (Figure 5). PGSS drying showed promising results for drying of green tea extracts because of low drying temperatures and an oxygen free atmosphere. The results showed that powders with different water and polyphenol contents could be produced by changing the process parameters.

## 3. Physicochemical Methods

### 3.1. Encapsulation by Cooling of Emulsions

This process consists of dissolving or dispersing the active compound in a melted wall material [41]. This melted phase is then emulsified in a continuous phase heated at a higher temperature than the melting point of the coating material. Usually, lipids having a low melting point such as Carnauba wax are used as coating agents. Then, the environment is suddenly cooled and particles solidify. The process allows the microencapsulation of hydrophilic or lipophilic molecules if a continuous phase is chosen for which these molecules do not have enough affinity.

A similar technique uses hydrophilic polymers capable of forming solid hydrogels during the cooling (gelatin, glucan or agarose).

A study showing the contribution of encapsulation on stability and antioxidant activity of four anthocyanin structures from an extract of black currant (BCAs) (delphinidin-3-*O*-glucoside, delphinidin-3-*O*-rutinoside, cyanidin-3-*O*-glucoside and cyanidin 3-*O*-rutinoside) was conducted [66]. In this work, the thermogelling polymer was ß-glucan. The effects of temperature, pH and presence of ferric and ferrous ions on the stability and activity of solutions of these four anthocyanins were studied. At the same time, a study of the release of anthocyanins encapsulated within glucan beads or cubes after various drying treatments was undertaken. The release of anthocyanins was more significant with the cubic forms, and the loss of activity of the encapsulated BCAs after treatment was comparable to that of the free anthocyanins. However, the antioxidant activity of the BCAs was more stable with regard to the free elements. Freeze-drying seemed to be less deleterious than infrared drying.

Epigallocatechin gallate (EGCG) was immobilized on lipid-coated nanoparticles [67]. EGCG protected in this way kept up to 90% of its capacity to stimulate the α-secretase *in vitro*, and the EGCG bioavailability after encapsulation was increased twice-fold compared to that of the free form *in vivo*.

Quercetin was shown to be 100 times more soluble when encapsulated on lipid-coated nanoparticles and stable for more than ten weeks, no degradation product being detected [68].

### 3.2. Emulsification-Solvent Removal Methods

**Figure 6.**
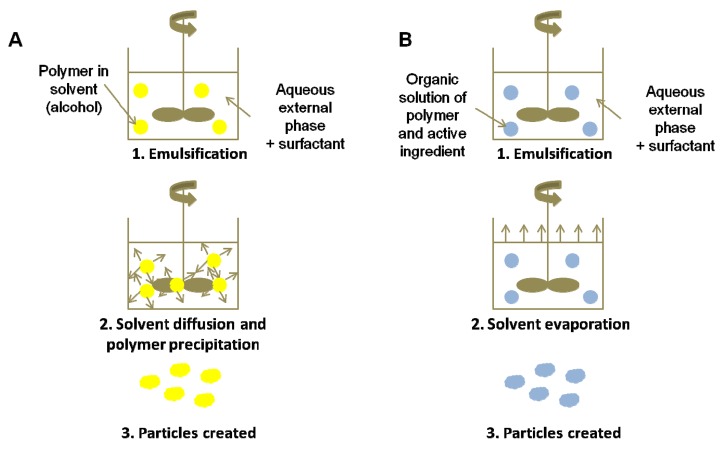
Encapsulation by (**A**) Emulsion/Extraction and (**B**) Emulsion/Evaporation methods.

These processes are based on evaporation or extraction of the internal phase of an emulsion giving rise to the precipitation of the polymer coating, first dissolved into this phase, in the form of particles (Figure 6).

The polymer intended to constitute the particle matrix is first dissolved in an organic solvent.

In the solvent evaporation method, a volatile solvent presenting a very low miscibility with water, such as dichloromethane, is chosen. Then, the active compound is dissolved or dispersed in the polymer solution. The mixture is emulsified very finely (ultrasounds, homogenizer) in a large amount of water containing surfactants, to obtain an oil-in-water (O/W) emulsion. Evaporation of the solvent is realized upon heating and/or under vacuum and with gentle stirring. This process is not recommended for the encapsulation of volatile compounds or molecules presenting a higher affinity for the continuous phase. This method can apply to the preparation of micro- or nanoparticles.

In the solvent extraction method, also known as nanoprecipitation, the solvent must be miscible with water in all proportions. The polymer solution containing the active compound is injected under agitation into a continuous aqueous phase containing a surfactant. Nanoparticles are formed by spontaneous diffusion of the solvent in the aqueous phase: the polymer, insoluble in the mixture of water and solvent, precipitates to form nanoparticles while trapping the active ingredient.

After removal of the solvent, particles are washed, collected by filtration or centrifugation, then dried or freeze-dried.

The operation speed directly influences the characteristics of the particles. Extraction of the solvent, faster than its evaporation, can lead for example to the formation of porous microspheres. Mainly on the nanometric scale, applications of the method to plant extract encapsulation are commonly found in scientific literature. The Table 2 below, far from being exhaustive, correlates some of these works.

### 3.3. Methods Based on Ionic Interactions

#### 3.3.1. Ionic Gelation

The ionic gelation process consists of extruding an aqueous solution of polymer through a syringe needle or a nozzle, in which the active material is dissolved or dispersed. Droplets are received in a dispersant phase and are transformed, after reaction, into spherical gel particles [41]; as is the case, for example, with sodium alginate used with a dispersant phase of calcium chloride.

Chitosan nanoparticles (carboxymethyl and chitosan hydrochloride) immobilizing a tea polyphenol extract [82] and an *Elsholtzia splendens* extract [83] were prepared this way. Particles showed to be good nanosystems for slow drug release by diffusion, the polyphenolic material being maintained active.

Catechins are powerful natural antioxidants but the major drawback is that they are very unstable in alkaline conditions encountered in biological fluids, and in some experimental protocols. That is why research teams studied encapsulation to bypass this limit to the application [84]. Catechin and (−)-epigallocatechin were immobilized within chitosan tripolyphosphate nanoparticles. After 24 hours, the measured antioxidant activity was 88.3% and 73.4%, respectively. After 24 hours, 50% of the encapsulated catechin was degraded, while 8 hours were enough to degrade the same amount of free catechin. On the other hand, epigallocatechin was much more unstable because after 40 min, more than a half was denatured.

A comparative study to demonstrate benefits (protection, stability, release) brought by the encapsulation of a yerba mate extract was undertaken [85]. Two models were tested: chitosan tripolyphosphate nanoparticles (ionic gelation) and microspheres prepared by spray-drying. Cosmetological and nutraceutical applications are evoked.

In the literature, calcium alginate gel beads containing an aqueous extract of *Piper sarmentosum* can also be found [86], and calcium pectinate gel beads immobilizing resveratrol for which loading was higher than 97%, with a total release of active ingredient after 10 hours [87].

#### 3.3.2. Acidic Precipitation

An extract of China green tea was successfully encapsulated either in sodium caseinate or in calcium caseinate beads by acid precipitation of the casein [88]. Among the beads, calcium-caseinate beads loaded with polyphenolic extract showed the best antioxidant properties.

#### 3.3.3. Complex Coacervation

This method is based on the ability of cationic and anionic water-soluble polymers to interact in water to form a liquid, neutral, polymer-rich phase called coacervate [41].

The complex coacervation process is the separation of an aqueous polymeric solution into two miscible liquid phases: a dense coacervate phase and a dilute equilibrium phase. The dense coacervate phase wraps as a uniform layer around dispersed core materials. Complex coacervation can start spontaneously upon the mixing of oppositely charged polyelectrolytes in aqueous media. The charges must be large enough to induce interaction, but not too large to cause precipitation.

The technology parameters are the pH, the ionic strength, the temperature, the molecular weight and the concentrations of the polymers.

The core material, which can be oily droplets, is dispersed into the aqueous solution of the two polymers. A change is made in the aqueous phase (pH) to induce the formation of a polymer rich phase that becomes the wall material. The coacervates are usually further stabilized by thermal treatment, crosslinking or desolvation techniques.

Basically, a complex coacervation process consists of three steps: formation of an oil-in-water emulsion, formation of the coating and stabilization of the coating.

According to this technique, the resulting particles are not perfectly spherical, and production cost is very high. However, the method is useful for the encapsulation of high value active molecules or for unstable substances, as is the case for polyphenols.

**Figure 7.**
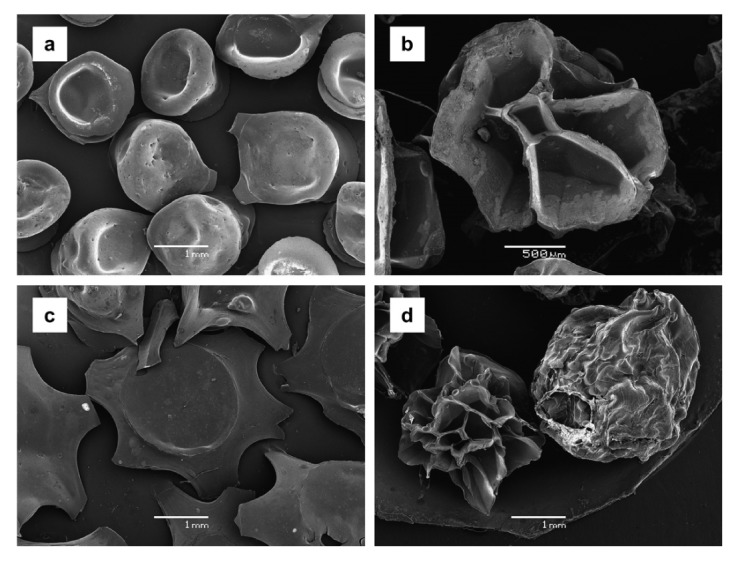
SEM microphotographs of control (**a**) dried in oven and (**b**) lyophilized beads; and alginate–chitosan (**c**) dried in oven and (**d**) lyophilized beads. Reprinted with permission from Elsevier [89].

A yerba mate (*Ilex paraguariensis*) freeze-dried extract containing 62.11 ± 1.16 mg of gallic acid per gram, was encapsulated using two different processes: firstly by ionic gelation (calcium alginate), and then by complex coacervation between calcium alginate and chitosan [89]. These two types of beads were shown to be resistant to oven-drying and freeze-drying (Figure 7). An antioxidant activity, higher than 85%, of the phenolic compound immobilized within the alginate beads was obtained, while for alginate beads coated with chitosan, only 50% of the polyphenol activity was preserved. In the study, the release of gallic acid revealed the influence of the nature of the wall material on the release of natural antioxidants present in yerba mate.

Propolis, a polyphenol-rich mixture collected by bees from some plants, possesses well-known therapeutic virtues. However, its use as a food additive is limited because of its solubility only in alcohol and its pronounced taste. The encapsulation of propolis using complex coacervation with pectin and soy protein appeared as an interesting alternative [90]. The result is a powder, easily dispersible in liquids other than alcohol, with indisputable antioxidant and antimicrobial properties, and from which the release of the active material can be controlled.

#### 3.3.4. Layer-by-Layer Process

**Figure 8.**
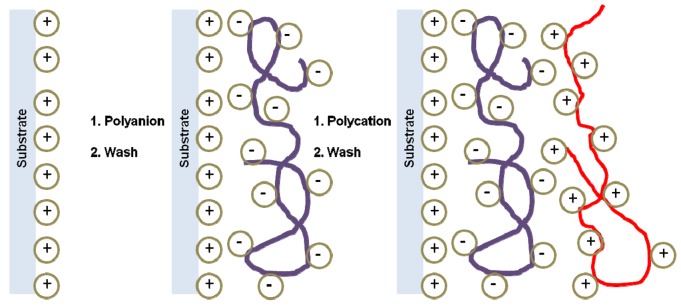
Schematic representation of polyelectrolyte self-assembling.

The Layer-by-Layer method (LbL) [91,92] consists of depositing alternating layers of oppositely charged materials onto mineral or organic substrates which constitute the core of the particle (Figure 8). In other words, it is a self-assembly technique based on electrostatic attraction of charged polymers leading to the formation of membranes of controllable thickness according to the number of stacked layers.

**Figure 9.**
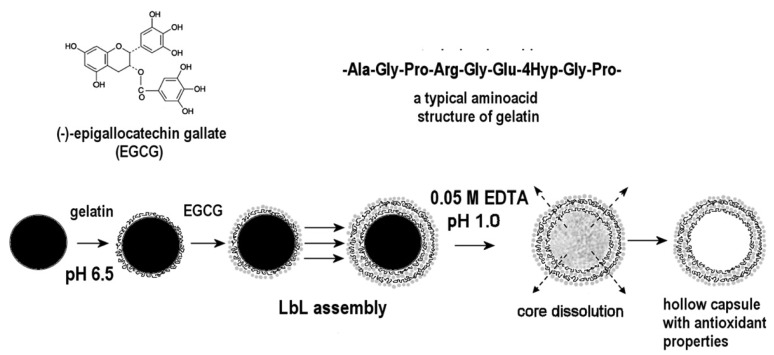
Scheme of gelatin A/EGCG hollow capsule preparation. Reprinted with permission from Elsevier [93].

Protein/polyphenol microcapsules with (−)-epigallocatechin gallate as a phenolic compound and type A gelatin as a protein source were obtained by this method [93] (Figure 9). The core of these particles was manganese carbonate, the shell being formed by polyelectrolytes assembled in successive layers (polystyrene sulfonate/polyallylamine hydrochloride, polyglutamic acid/poly-L-lysine, dextran sulfate/protamine sulfate, carboxymethyl cellulose/gelatin A) into which the EGCG was inserted according to the LbL assembly method. Synthesis, characterization and release studies of polyphenols from these particles revealed that EGCG inside the membrane preserved its antioxidant activity and blocked the production of hepatocyte growth factor (HGF) from cancer cell lines MBA-MD-231 as effectively as free EGCG.

The same idea was applied by this team to the preparation of gelatin nanoparticles, coated by the LbL method and loaded with EGCG. The resulting nanoparticles showed an interesting inhibitory effect on HGF-induced cell scattering [94].

### 3.4. Methods Based on Hydrophobic Interactions

#### 3.4.1. Micelles

In an aqueous solution, amphiphilic polymers are able to self-organize into supramolecular arrangements possessing a hydrophobic central core and a hydrophilic crown [95]. As for surfactant micelles, these structures can appear if the polymer concentration in solution is higher than the critical micellar concentration (CMC). To improve the stability and reduce the polydispersity of polymeric micelles, a crosslinking is generally realized. After formation of micelles, the crosslinking can take place either in the core or in the hydrophilic part.

In a study of Lu *et al.* [96], polycaprolactone (PCL) constitutes the hydrophobic core and poly(ethylene glycol) (PEG) is the hydrophilic shell of micelles. These resveratrol-loaded nanoparticles showed a protective effect of PC12 cells against superoxide-induced damage during the phenomenon of oxidative stress.

Artemisinin and curcumin are two molecules that can exert a potential synergistic antimalarial action but they present a poor solubility in aqueous environments, limiting their use. Their immobilization in micelles of sodium dodecyl sulphate (SDS) combined solubilisation with protection against oxidation phenomena [97].

Polyphenolic fractions from witch hazel (*Hamamelis virginiana*), particularly rich in galloyl groups, in SDS-micelles were able to effectively protect α-tocopherol (α-TOH) through reduction of the α-tocopheroxyl radical [98].

Dr Ray's team has used nanotechnology to protect isolated curcumin in tiny particles more easily absorbed into the bloodstream. In this work, a micelle-forming polymer that encapsulates curcumin in its hydrophobic core (NanoCurc^®^) is used. This copolymer of *N*-isopropylacrylamide (NiPAAm), *N*-vinyl-2-pyrrolidone and poly(ethylene glycol) acrylate spontaneously forms micelles in water. The nanocurc^®^ curcumin micelles can potentially reach the brain in much higher concentrations than free curcumin. The study showed fewer signs of inflammatory damage both in cell cultures (SK-N-SH cells) and in animal models of Alzheimer's disease. The aim of the next step will be to demonstrate that nanocurc^®^ will do what curcumin could not during the inevitable upcoming clinical trial [99]. A systemic administration of these nanoparticles blocked tumor growth and metastases in preclinical models of pancreatic cancer with a significant reduction in the activation of the nuclear factor-kB, and in the expression of the matrix metalloproteinase-9 and of the cyclin D1.

While encapsulating curcumin is a way to increase plasma and tissue concentrations of the active agent, the choice of nanomaterial used in these formulations must allow the controlled release of the compound directed at the target tissue. Earlier work in a mouse model of pancreatic cancer demonstrated the inhibition of tumor growth when Nanocurc^®^ was administered parenterally. For the treatment of neurological cancers the blood brain barrier represents another potential barrier to Nanocurc^®^'s therapeutic effectiveness [100]. However, a recent report evaluating the therapeutic potential of Nanocurc^®^ in an animal model of Alzheimer's disease did demonstrate significant accumulation of curcumin in brain tissues when administered via I.P. injection, suggesting that Nanocurc^®^ could be useful in treating neurological cancers [99].

#### 3.4.2. Liposomes

**Figure 10.**
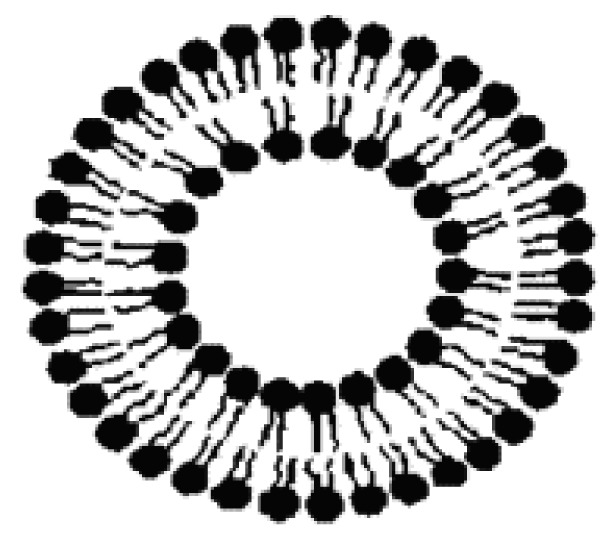
Schematic representation of a liposome.

Described and synthesized for the first time in 1965, liposomes are artificial vesicles formed by one or more concentric lipid bilayers separated by water compartments (Figure 10).

Liposomes are often distinguished according to their number of lamellae and size. Small unilamellar vesicles (SUV), large unilamellar vesicles (LUV) and large multilamellar vesicles (MLV) or multivesicular vesicles (MVV) are the main classes.

Due to their structure, liposomes are used to target, protect, release, immobilize or isolate hydrophilic, lipophilic or amphiphilic substances.

For the production of liposomes, many methods are available [101]. The most classical method, realized by hydration of dried phospholipid films, is the famous Bangham method [102], named after its inventor, Alec Bangham, who passed away very recently.

In spite of a dense literature dealing with liposomes, these vectors present restrictive limitations of use [103]. The first drawback is their instability in biological fluids and the high speed of active ingredient release. Another disadvantage of liposomes is that their payload of the active ingredient can only be low. Furthermore, they are unstable upon storage. However, the most worrying disadvantage in terms of industrial production is related to low reproducibility.

A comparative study has revealed that the encapsulation efficiency of phenolic compounds depends on the morphology of the liposome, itself dependant on the method implemented for their preparation [104]. The interaction of plant polyphenols, in particular tea catechins, with the lipid bilayer of liposomes depends on the structure of the polyphenol and on external factors such as salt concentration [105].

The encapsulation of two isomers, separately in liposomes of the same nature (+)-catechin and (−)-epicatechin, showed comparable encapsulation efficiencies and release kinetics [106]. On the other hand, another type of catechin, EGCG, showed a better encapsulation rate. The difference between these three molecules is that EGCG possesses in its structure a galloyl group which confers a higher lipophilicity.

The optimized formulation of a tea polyphenols-vitamin E complex in liposomes prepared by the reverse-phase evaporation method allowed to effectively encapsulate these compounds, leading to their protection, an increase of the solubility of the vitamin E, and favoring the transdermal penetration of the complex [107].

Brown algal phlorotannins encapsulated in unilamellar vesicles prepared by the extrusion method, maintained their activity, *i.e.*, lipid peroxidation inhibition and radical scavenging activities [108].

The demonstration that liposomes improve the bioactivity and the bioavailability of polyphenols is reported in many papers. Possessing very powerful antioxidant properties, curcumin is a much appreciated polyphenolic pigment, in particular for applications in the field of human health. The properties of curcumin as an anti-HIV, anticancer, antioxidant and anti-inflammatory drug have been demonstrated. However, this weakly water soluble and particularly unstable compound is poorly absorbed by human gastrointestinal tract after oral administration.

To improve its bioavailability and functionality, curcumin was encapsulated in lecithin liposomes (LEC) [109]. The resulting liposomes were small unilamellar vesicles of 263 nm in size, with an encapsulation efficiency of 68%. Oral administration of the LECs favored the intestinal absorption of curcumin, leading to an important increase of the plasma antioxidant activity.

Another example is the study of quercetin-loaded liposomes which, by intranasal administration, presented anxiolytic and cognitive beneficial effects in comparison with the native quercetin taken orally [110,111].

Other examples of applications of liposomes loaded with polyphenols are compiled in Table 3.

Other examples of the use of micelles or liposomes for the delivery of polyphenols can be found in a recent review [122].

## 4. Chemical Methods

### 4.1. In Situ Polymerization

Mainly used for the synthesis of nanocomposites, the *in situ* polymerization process consists of emulsifying the monomer component, mostly vinylic and acrylic compounds such as styrene or methyl methacrylate, in an aqueous phase added with an appropriate surfactant. The polymerization having been started, the resulting water-insoluble polymer gives microspheres [41].

A recent article mentions the possibility of encapsulating quercetin by *in situ* polymerization [123]. The influence of the reaction parameters was studied. The paper reveals the interference caused by the presence of quercetin within the methyl methacrylate solution on the polymerization reaction speed and quality. The presence of ascorbic acid favored the polymerization reaction and decreased the oxidation of immobilized quercetin.

### 4.2. Interfacial Polycondensation and Interfacial Cross-Linking

**Figure 11.**
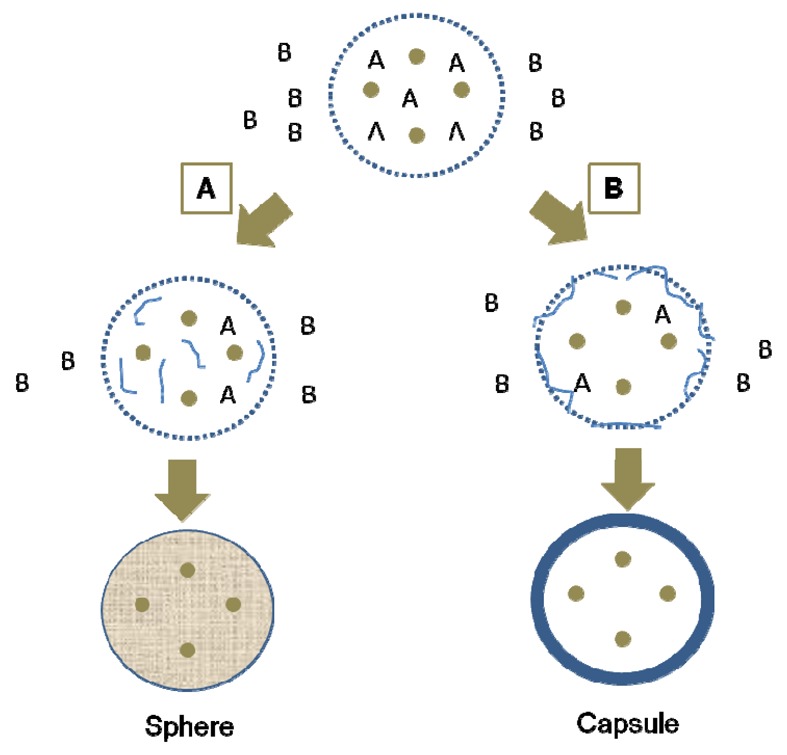
Principle of the microencapsulation by interfacial polymerization. (**A**) The oligomer is soluble in the droplet; (**B**) the oligomer is insoluble in the droplet.

Interfacial polycondensation is a chemical reaction by which a membrane made of polymers is created around emulsion droplets [124]. The reaction takes place at the interface between the continuous and dispersed phases. In the emulsion, each phase contains a type of monomer (Figure 11). This process can be applied to aqueous or organic active materials. In the case of a water-soluble active ingredient, the process takes place as follows: a solution containing the active compound and a water soluble monomer A is prepared with distilled water; an oil-in-water (W/O) emulsion is formed by emulsification of the aqueous phase in an organic external phase; then, the organosoluble monomer B is added to the organic phase; finally, interfacial polycondensation reaction between the two monomers at the O/W interface is started. The method can also apply to an organic active material in an organic solution. In this case, using the same process, the interfacial polycondensation reaction is conducted in a water-in-oil (O/W) emulsion.

Two situations can occur: If the oligomer is soluble in the droplets (Figure 11A), a polymeric matrix creates inside the droplets and microspheres are thus formed. If the oligomer is insoluble in the droplets (Figure 11B), a polymeric membrane is formed around them, and the droplets are thus individually encapsulated by the polymer. This leads to the formation of reservoir microcapsules.

Formulation is based on a large number of parameters: the nature of monomers, the nature and concentration of the surfactant used, the properties of solvents, the physical parameters of the stirring (speed, time, type of mobile), each of these parameters influencing the membrane properties and the size distribution of the particles.

However, unusual chemical reactions between the immobilized active compound and monomers can take place. The solubility of the active compound in solvents can be a drawback. The use of potentially toxic monomers can be limiting in this encapsulation process, particularly for biomedical applications.

**Figure 12.**
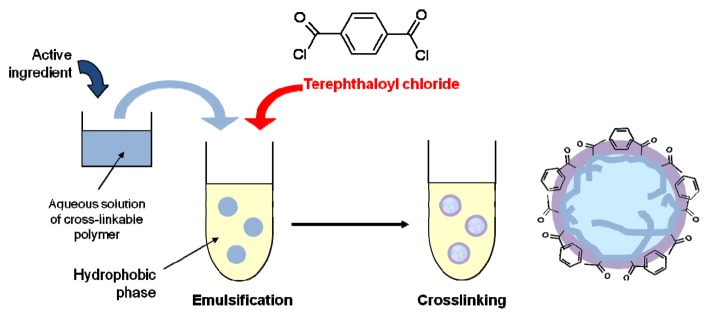
Mechanism of microcapsule formation by interfacial cross-linking of a hydrosoluble polymer, involving terephthaloyl chloride as an organo-soluble cross-linking agent.

When the water-soluble monomer is replaced by an oligomer or polymer, this is known as interfacial cross-linking (Figure 12). In this case the condensation reaction involves the reactive groups of the bifunctional organosoluble monomer and the functional groups of the water soluble oligomer or polymer.

**Figure 13.**
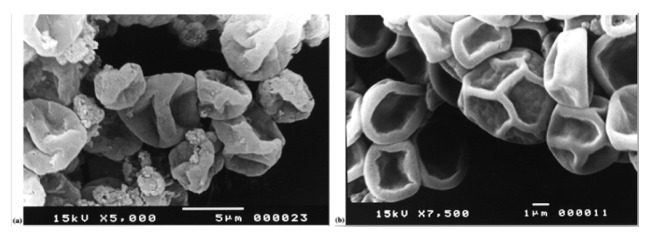
Scanning electron micrographs of proanthocyanidin microcapsules (**a**) prepared at pH 9.8; (**b**) prepared at pH 11. Reprinted with permission from Elsevier [125].

Microparticles made of cross-linked grape proanthocyanidin (GPO) were developed using this method [125]. In these microcapsules, the polyphenolic compound constitutes the membrane material, and the cross-linking reaction stabilizes the molecule while maintaining a radical-scavenging activity. The cross-linking reaction of GPO with terephthaloyl chloride (TC) involves hydroxyphenolic groups leading to the establishment of ester bonds that were detected by infrared spectroscopy. Cross-linked GPO microcapsules, obtained at pH 9 and 11, had a size lower than 10 μm (Figure 13) and were stable for more than five months at 45 °C in an aqueous environment. The microcapsules were slowly degraded in plasma and presented an interesting antioxidant activity, although slightly lower than the initial GPO. The method of preparation of these microcapsules by interfacial cross-linking of polyphenols is patented [126].

## 5. Other Stabilization Methods

### 5.1. Encapsulation in Yeasts

The encapsulation of essential oils and aromas using yeast cells (*Saccharomyces cerevisiae*) as the encapsulant material turned out to be not only cheap but also very effective in terms of loading [127].

The permeability of the cell membrane ensures an active diffusion, and effectively protects against evaporation or oxidation phenomena. This method is typically used for the encapsulation of small lipophilic molecules as found in essential oils. Recently, the method was adapted to the encapsulation of water soluble polyphenols.

Chlorogenic acid was encapsulated in baker's yeast cells [128]. According to the technique described by Bishop *et al.* [127], cells were emptied out of their content by autolysis using a plasmolyzing agent (NaCl 5%).Then, the empty cells were dispersed in an aqueous phase containing chlorogenic acid, and loaded by re-swelling in this solution. The encapsulation efficiency was around 13 %. The encapsulation increased the stability of the active compound towards a thermal and hydric stress, whereas it did not hinder the *in vitro* release. New works led by Paramera *et al.* showed that the stability and release properties of curcumin encapsulated in *Saccharomyces cerevisiae* offers a better thermal protection (200 °C) than ß-cyclodextrins or spray-drying with modified starches [129].

### 5.2. Co-Crystallisation

This process consists of introducing the aromatic compound into a saturated solution of sucrose (syrup). The spontaneous crystallization of this syrup is realized at high temperatures (above 120 °C) and with a low degree of humidity. The crystal structure of sucrose is modified, and small crystal aggregates (lower than 30 μm) trapping the active molecule are formed.

The main advantages of the co-crystallization technique are that the granular product obtained possesses a very low hygroscopicity, a good fluidity, and a better stability. Furthermore, the co-crystallization offers a good economic alternative and remains a flexible technique because of its simplicity.

The encapsulation of a yerba mate extract containing caffeic acid derivatives and flavonoids was successfully realized by co-crystallization in a saturated sucrose solution [130]. The resulting crystals had a size between 2 and 30 μm. The co-crystallization significantly reduced the hygroscopicity of the yerba mate extract without affecting its high solubility. The process thus appears as a promising alternative for the preservation of phenolic compounds in future industrial applications.

However, only a few studies deal with encapsulation of polyphenols by this process.

### 5.3. Molecular Inclusion

Molecular inclusion classically appeals to cyclodextrins (CDs). CDs represent a family of cyclic oligosaccharides consisting of glucopyranose subunits bound through α-(1,4) links. These natural products resulting from starch degradation by bacterium *Bacillus macerans* were discovered in 1891 by Villiers. Three families are mainly used or studied: α-, β- and γ-cyclodextrins, composed of 6, 7 or 8 subunits, respectively. These cyclic molecules possess a cage-like supramolecular structure, able to encapsulate various guest molecules [131–133].

A large number of weakly water-soluble molecules were trapped in cyclodextrins: resveratrol in β-CD and maltosyl-β-CDs [134], olive leaf extract in β-CD [135], kaempferol, quercetin and myricetin in 2-(hydroxy-propyl)-ß-cyclodextrines (HP-β-CD) [136], rutin in β-CDs [137], hesperetin in HP-ß CDs [138], 3-hydroxyflavone in α- and β-CDs [139]. The inclusion of these last ones within these cage-like structures led to an increase in their water solubility as well as in their antioxidant capacity.

Furthermore, the encapsulation rate of a phenolic compound is related directly to the type of CD used. For example, studies report that the encapsulation efficiency of curcumin in different CDs is variable [140,141]. Indeed, HP-ß-CD immobilizes more curcumin molecules in comparison with the other tested CDs. On the other hand, rosmarinic acid showed maximal inclusion ability in the compartment of methyl-ß-cyclodextrin (M-ß-CD) [142]. The quercetin and myricetin affinities for CDs are also related to the type of CD used [140].

CDs appeared to be good thermal protectors [143]. Indeed, a flavonoid-rich extract immobilized into ß-CD revealed a thermo-oxidative stability, as compared to the free extract which was totally oxidized in the same conditions. Perspectives of an application as a flavonoid-rich food complement or as a food additive are evoked.

The complexation of olive oil antioxidant hydroxytyrosol with ß-CDs or HP-ß-CDs was studied [144]. Only ß-CDs appeared to be very strong photo-protectors of polyphenolic compounds subjected to ultraviolet radiation (λ = 254 nm).

**Figure 14.**
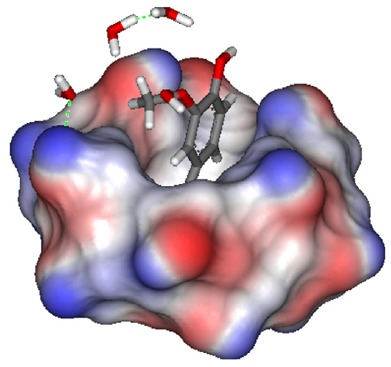
Molecular model of inclusion complex ferulic acid/α-CD. Reprinted with permission from Elsevier [145].

The encapsulation of ferulic acid within α-CDs improved the chemical stability and the bioavailability on the skin [145]. The structure of the inclusion complex of ferulic acid in α-CD was analyzed by modeling (Figure 14). This study showed that the insertion of the ferulic acid molecule within the lipophilic core of α-CDs involved the -COOH and the α,ß- unsaturated groups present on a part of the aromatic ring. This molecular encapsulation increases the ferulic acid photo-stability, and renders the polyphenol able to protect the skin against the sun's harmful ultraviolet rays.

### 5.4. Freeze-Drying

Freeze-drying, also known as lyophilization, is one of the most used processes for the protection of thermosensitive and unstable molecules. It is a dehydration operation at low temperature consisting in eliminating water by sublimation of the frozen product.

A polyphenol-rich raspberry (*Rubus chamaemorus*) extract was stabilized by freeze-drying, with two types of maltodextrins (DE 5-8 and DE18.5) as material coating [146]. The freeze-dried particles were stable over long periods and provided to polyphenols an effective protection against the oxidation phenomenon during their storage, whereas antioxidant activity remained identical.

Besides, a study led on a hibiscus anthocyanin extract showed that, free or co-freeze-dried with a pullulan matrix, its antioxidant activity after storage was unchanged. In this case, freeze-drying did not alter the properties of the free extract but brought no benefit to the antioxidant activity [147].

## 6. Conclusion

Polyphenols are among the most powerful active compounds synthesized by plants, and show a unique combination of chemical, biological and physiological activities. However, their limited stability and/or solubility, often combined with a poor bioavailability, have to be resolved in order to make these compounds more able to answer growing demands in cosmetics, nutrition and health. In this review, the results of recent studies implementing various encapsulation techniques applied to extracts and/or polyphenolic compounds from plants confirmed that encapsulation is an interesting means to potentialize their activity. Among them, spray-drying is the most common technique used to encapsulate polyphenols.

The functions provided by encapsulation to the final product must be clearly established in order to select the encapsulation process and the most suitable coating material. The different processing conditions through which the product will go before release are of essential consideration for the final activity, and exposure to oxidizing conditions like high temperatures, air, metal ions, will have to be avoided or shortened during the process. Furthermore, particle properties (composition, particle size and density, release mechanism and kinetics, degradation mechanism and kinetics, final physical form) may be changed by varying the processing parameters, in order to suit specific applications. Other important features to take into account are the optimum concentration of the active core, and the overall cost of the process.

The various research results reported in this paper revealed that encapsulation provided a significant protection against drastic conditions such as oxidation and thermal degradation, thereby contributing to increase the shelf life of the encapsulated active ingredient. Furthermore, encapsulation was also shown to mask an unwanted flavor, smell or taste, to control the release, to change the physical properties of the initial material, and to improve the bioavailability of the polyphenolic compound. Interesting results from cell culture studies and animal models have also been obtained. The challenge to convert the most powerful polyphenols into usable compounds has then been resolved through innovative formulations.

In this domain, the progress should accelerate the reasoned use of natural polyphenolic compounds, not only as food additives or as nutritional supplements, but also as active cosmetics or as drugs. There is no doubt that, boosted by recent remarkable scientific advances, human clinical trials will soon be in progress.

## Figures and Tables

**Table 1.** Main classes of plant polyphenols, structures, sources, their specifications and biological properties [2,3,38–40].

	**Carbon skeleton**	**Examples**	**Sources**	**Specifications**	**Main biological properties**
**Phenolic acids and coumarines**					
Hydroxybenzoic acids	C6-C1	Gallic acid, Vanillic acid, Protocatechuic acid, *p*-Hydroxybenzoic acid	Tea Red fruit (raspberry, black currant, strawberry)	Very common, in free form as well as combined, not much studied and not considered to be of great nutritional interest, sensitive to temperature, oxidation, light and pH, water soluble	Very limited therapeutic interest, antimicrobial activity and fungitoxicity, anti-inflammatory properties of salicylates
Hydroxycinnamic acids	C6-C3	Caffeic acid, *p*-Coumaric acid, Sinapic acid Ferulic acid	Fruit (kiwis, blueberries, apples) Cereal grains (wheat, rice, oat flours)	Rarely found in free form, often esterified, sensitive to oxidation and pH, slightly soluble in water	
Coumarines		OmbelliferoneAescu letin, Scopoletin	Tonka bean, bark (chestnut), medicinal plants (*Melilotus officinalis*, *Angelica officinalis*)	Free coumarines are soluble in alcohols and organic solvents, the heterosidic forms are less soluble in water	Anti-inflammatory and antiviral activities, limited pharmacological applications: hepatotoxicity
Stilbenes	C6-C2-C6	Resveratrol	Medicinal plants (vine)	Found only in low quantities in the human diet	Anticarcinogenic effects, anti-inflammatory activity
**Flavonoids**	C6-C3-C6				
Flavonols		Myricetin, Quercetin, Kaempferol and their glycosylated forms	Fruit and vegetables (Onions, curly kale, leeks, broccoli, blueberries), red wine and tea	Flavonols are the most ubiquitous flavonoids in food	Vitamin P factor protecting capillaries and veins, often anti-inflammatory, antiallergenic, antiviral, anti-spasmodic, antibacterial, antioxidant and anti-carcinogenic properties, hepatoprotector, some are powerful enzymatic inhibitors
Flavones		Aspigenin, Luteolin, Tangeretin, Nobiletin, Sinensetin	Parsley, celery, cereals (millet and wheat) Skin of citrus	Flavones are much less common than flavonols in fruit and vegetables	
Flavanones		Hesperetin, Naringenin, Eriodictyol	Citrus fruit (grapefruit, orange, lemon), tomatoes and some aromatic plants (mint)	Sensitive to oxidation, light and pH, bitter taste	
Isoflavones		Genistein, Daidzein, Glycitein	Leguminous plants (soya and its processed products)	Structural similarities with estrogens confers pseudohormonal properties	
Flavanols					
Monomer form		Catechin, Epicatechin	Fruit (apricot, cherry, grape, peach, apple), green and black tea, red wine and cider	Sensitive to oxidation, light and pH, astringent and bitter taste, slightly soluble in water	
Polymer form Proanthocyanidins	(C15)n	Castalin, Vescalin	Fruit (grapes, peaches, kakis, apples, berries), beverages (wine, cider, tea, beer), chocolate	Responsible for the astringent character and bitter taste, sensitive to high temperature and oxidation, water and alcohol soluble	
Anthocyanins		Cyanidin, Pelargonidin, Delphinidin, Petunidin	Red wine, some varieties of cereals, some leafy and root vegetables (aubergines, cabbage, beans, onions, radishes), flowers and most abundant in fruit	Plant pigments, highly sensitive to temperature, oxidation, pH and light, water soluble	
**Lignans**	(C6-C3)_2_	Pinoresinol, Podophyllotoxin, Steganacin	Flax seed, sesame seed, cereals (rye, wheat, oat, barley), cruciferous vegetables (broccoli, cabbage), and fruit (apricots, strawberries)	One of the major classes of phytoestrogens, relatively stable under normal conditions, water soluble, unpleasant flavour	Hepatoprotector, antimitotic, antiviral, antihypertensive and cytostatic activities, inhibitors of enzymatic reactions

**Table 2.** Emulsification-solvent removal methods applied to polyphenol encapsulation. PLA: Polylactide; PLGA: poly (lactic-co-glycolic) acid; PMMA: polymethyl methacrylate; DMAB: dimethylaminoborane; EC: ethylcellulose; PEG: polyethylene glycol; PCL: polycaprolactone; EE: eudragit; PVA: polyvinylic alcohol; PBS: phosphate-buffered saline.

**Encapsulation methods Nature of matrix**	**Polyphenols**	**Observations**	**References**
**Solvent evaporation**			
PLA	Quercitrin (*Albizia chinensis*)	Increase in the size of empty nanoparticles, antioxidant activity 40%, *in vitro* slow release in PBS, molecular stability, oral administration, therapeutic and nutraceutic applications.	[69]
	Quercetin	Nanoparticles increase in size, antioxidant activity 96.7%, slow and total release after 72 hours, potential therapeutic applications.	[70]
PLGA	Curcumin	Nanoparticles, lengthen retention time in the body and improve bioavailability, oral bioavailability of curcumin encapsulated was 22-fold higher than free curcumin, absorption of curcumin was significantly increased by nanoformulation.	[71]
	Epigallocatechin gallate (EGCG)	Biodegradable nanoparticles, free molecule having low biopharmaceutical and pharmacokinetic properties, clinical development, load efficiency of 70 %, antioxidant efficiency estimated *in vivo*, EGCG encapsulated oral administration *versus* solution EGCG administered parenterally by injection at same concentration: acts 3 times more quickly, therapeutic applications.	[72]
	Ellagic acid	Nanoparticles, two stabilizers are tested (DMAB and PVA): influence on the size, loading, release kinetics in PBS, stability, cytotoxic activities, *in situ* intestinal permeability, are estimated.	[73]
PMMA	PEG, riboflavine-5′-phosphate	W/O/W double emulsion, microparticles, protective role of the membrane on its photosensitive contents is demonstrated.	[74]
PLGA-PCL	Ellagic acid	Nanoparticles, two stabilizers are tested (DMAB and PVA): influence on the size, loading, release kinetics in PBS, stability, cytotoxic activities, therapeutic applications, oral administration of smaller quantity for comparable effect *versus* free.	[75]
EC	Tea polyphenol (TP)	Microcapsules, formulation conditions are investigated, two stabilizers are tested (DMAB and PVA), TP not denatured by the process, release kinetics and stability satisfactory.	[76]
	Bayberry polyphenols	Microcapsules, the antioxidant activity of bayberry polyphenols could be effectively protected, smooth surface shape with a particle size distribution of 10–97μm, storage stability of bayberry polyphenols against adverse environment was also remarkably improved by microencapsulation, release rate of bayberry polyphenol from microcapsules: 2.56–15.14% under simulated gastric fluid with pH of 2–6; and 87.37% under simulated intestinal fluid 24 with pH of 8.	[77]
Kafirine	Catechin, condensed tannins (*Sorghum*)	Microparticles, comparative study between particles loaded with catechin and those loaded with Sorghum, matrix chosen as its porosity, release kinetics study under gastric conditions, the encapsulation of these molecules does not affect particle size (5–6μm), but surface morphology is different: catechin particles have a porous hard surface and sorghum particles have an irregular shape, strongly aggregate, hard and smooth surface, stability over 4 hours: absence of degradation compounds but loss of antioxidant activity: 70% (Catechin) and 50% (Sorghum).	[78]
**Solvent extraction**						
PEG-PCL	Resveratrol	Nanoparticles, resveratrol involved in many cellular mechanisms, high load because very lipophilic molecule, low concentrations are enough for obtaining a high cytotoxicity *versus* free resveratrol, application in chemotherapy (ex: therapeutic strategy for malignant glioma).	[79]
EE-PVA	Quercetin (QU)	Quercetin-loaded nanoparticles (QUEN system), ratio QU:EE:PVA is 1:10:10, particles ± 85nm, size polydispersity, loading around 99%, various functionality tests reveal antioxidant activity more important for the QUEN.	[80]
PLA-PEG	EGCG	Chemoprevention, Nano-EGCG compared with free EGCG: *In vitro*: produced remarkably superior effects on proliferative ability in human prostate cancer PC3 cells with over 10-fold dose advantage→ nanoencapsulation removes the penetration barriers at cell surfaces; *In vivo* (xenograft model) : similar extent of tumor growth inhibition (10-fold lower dose of nano-EGCG was required), decrease of serum PSA by nano-EGCG best marker of prostate cancer in human.	[81]

**Table 3.** A variety of liposome techniques employed for the encapsulation of polyphenols.

**Polyphenols**	**Applications**	**Biological activities**	**Loading**	**Route of administration**	**References**
Curcumin	Photo-ageing attenuation (demonstration in mice)	Antioxidant, Anti-inflammatory Photo-protector		Oral	[112]
Resveratrol	Improvement of the cellular answer to oxidative stress via rapid and potent cellular internalization.	Antioxidant Photo-protector	>70%	*In vitro*	[113]
	Nano-sized vesicles, inclusion of resveratrol retarded drug release *in vitro*, this system was associated with no or poor liver and kidney toxicity *in vivo.*	Cardiovascular protector	≈70%	*In vitro* and *in vivo* Intraperitoneal injection	[114]
Quercetin	Reduced anxiety and cognitive functions, dose administered decrease, increase in circulation time, vectorization, increase in brain penetration efficiency.	Antioxidant, Anticancer	60%	Nasal	[111]
	Biodisponibility increased, vectorization, hepatic membrane penetration efficiency greatly improved.	Hepato-protector		Transdermic	[115]
Myrtle (*Myrtus communis*) extract	Antioxidant and antimicrobial activities superior to free forms.	Antioxidant, Antimicrobial		*In vitro*	[116]
Thyme (*Thymus sp.*) extracts					[117]
Silymarin	Biodisponibility increase.	Hepato-protector	>69%	Oral	[118]
Catechin	Skin penetration efficiency improved.	Chemo-protector, Antioxidant	>90%	Transdermic	[119]
Catechin, (-)-epicatechin and EGCG	Biodisponibility, EGCG encapsulated has tissue penetration ability improved *versus* 2 other catechins.	Antioxidant, Anticancer		Intratumoral	[106]
	Liposomes may influence drug deposition in tumor tissues.	Antioxidant, Anticancer		Topical application and intratumoral	[120]
Tea extract	Stability 4 °C increased.	Feasibility evaluation		*In vitro*	[121]
